# Supplementary figures and images for: Transcriptomic landscape and chromatin accessibility uncover pivotal regulators driving programmed larval-larval molting in the domesticated silkworm
Source: PLoS Genet. 2025 Aug 19;21(8):e1011837. doi: 10.1371/journal.pgen.1011837 (PMC12380352; doi:10.1371/journal.pgen.1011837)

Fig. S1

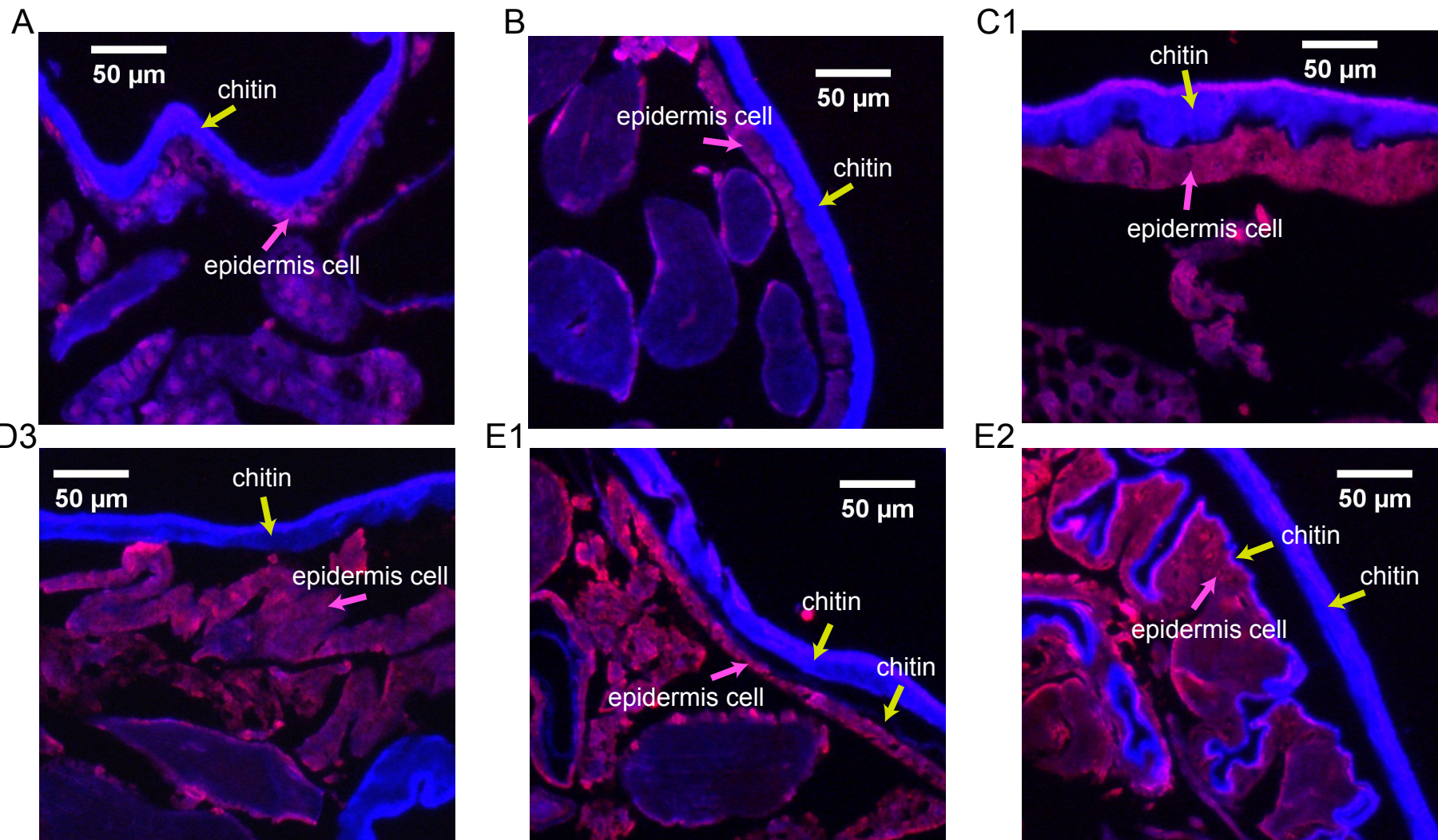

Supplement: S1 Fig — (S1_Fig.PDF) [file pgen.1011837.s001.pdf]

Fig. S2

a

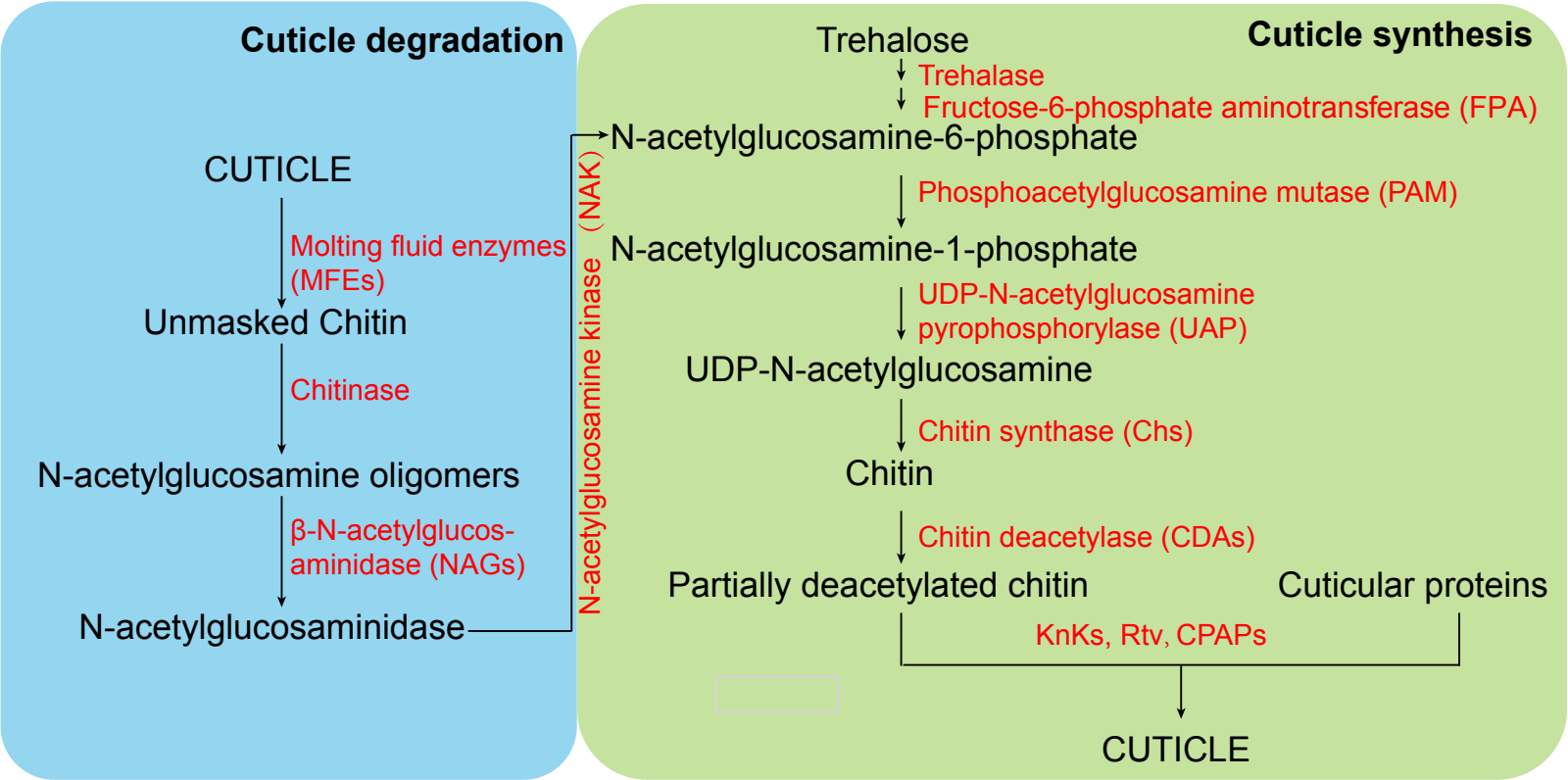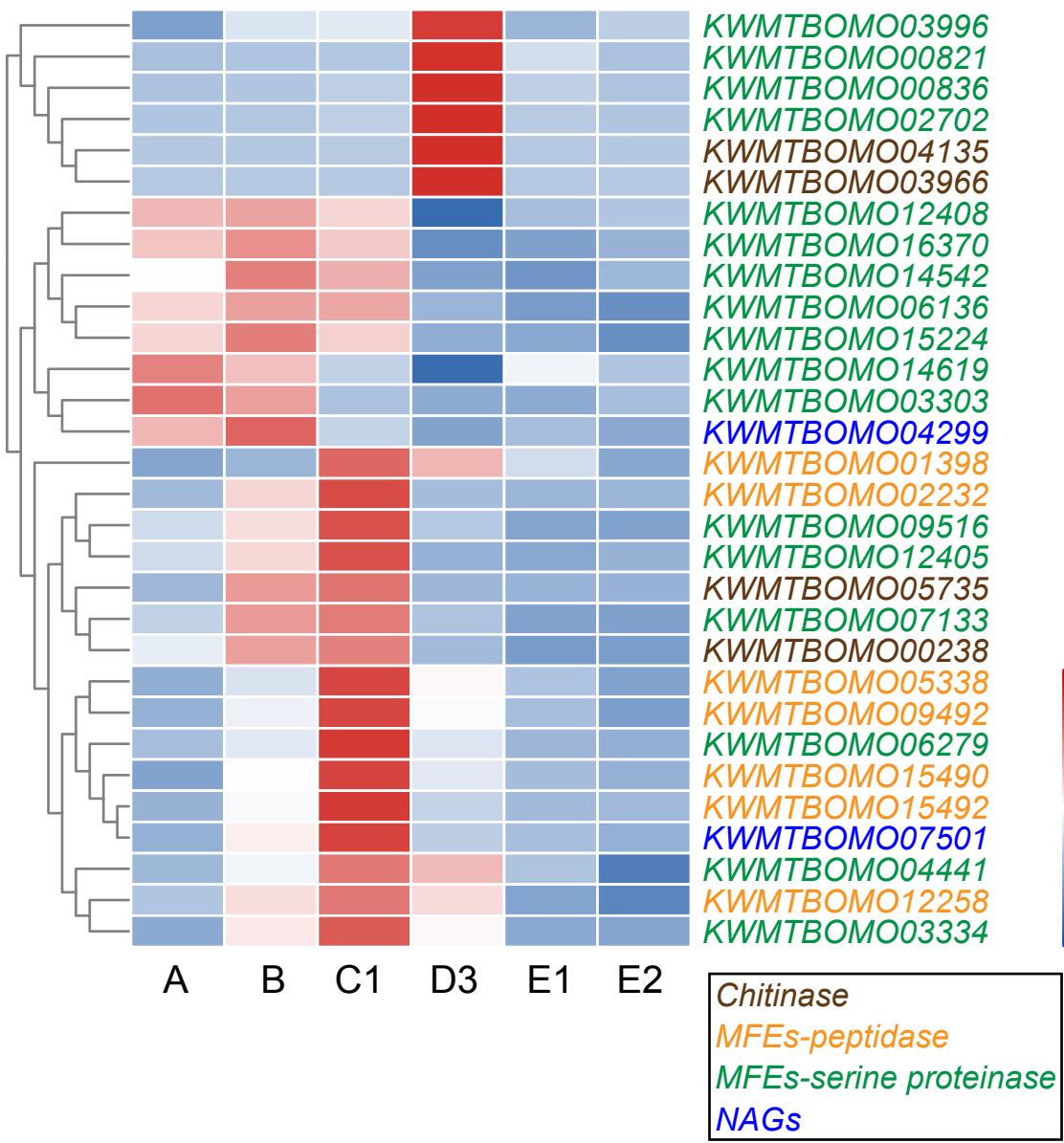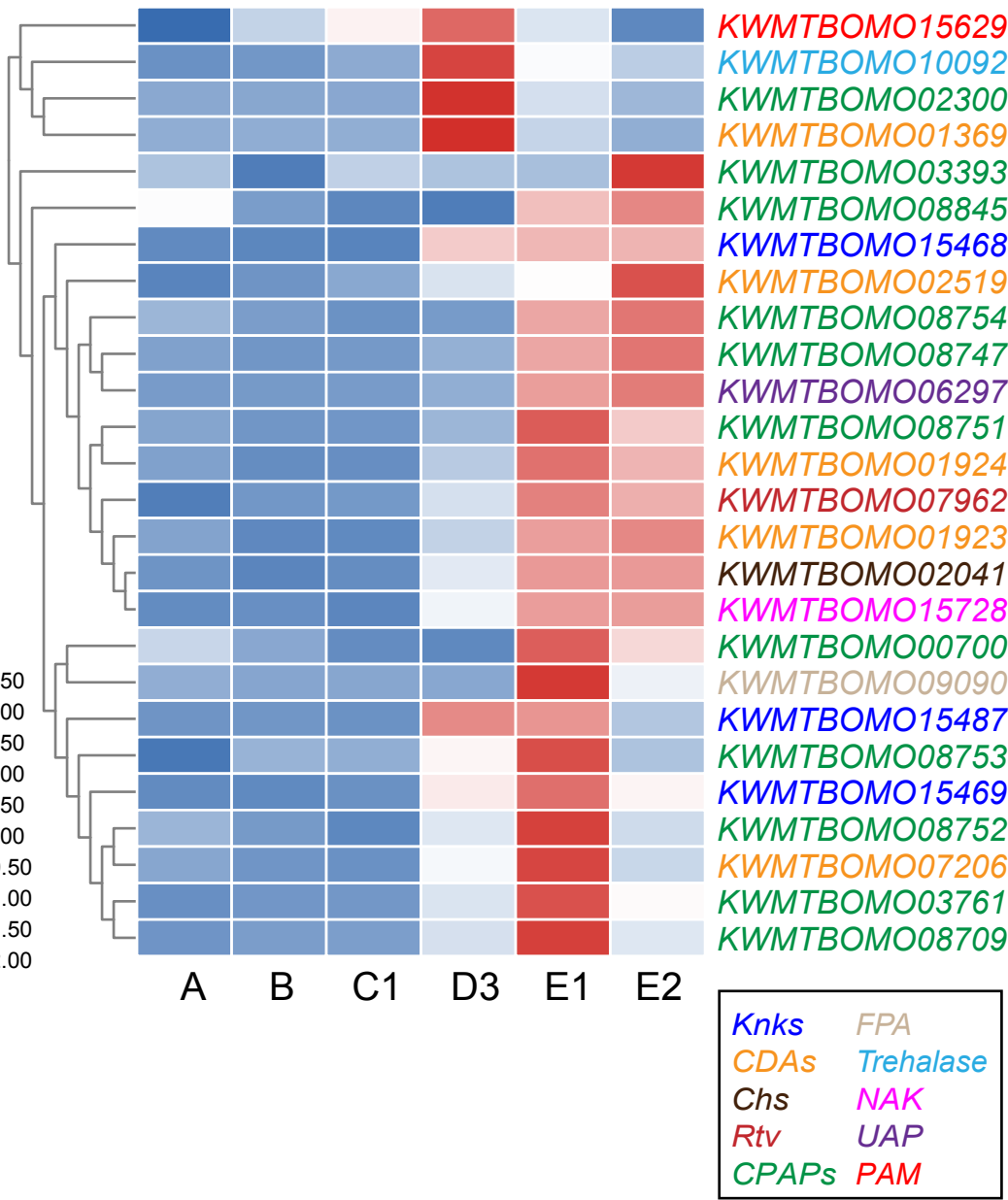

b

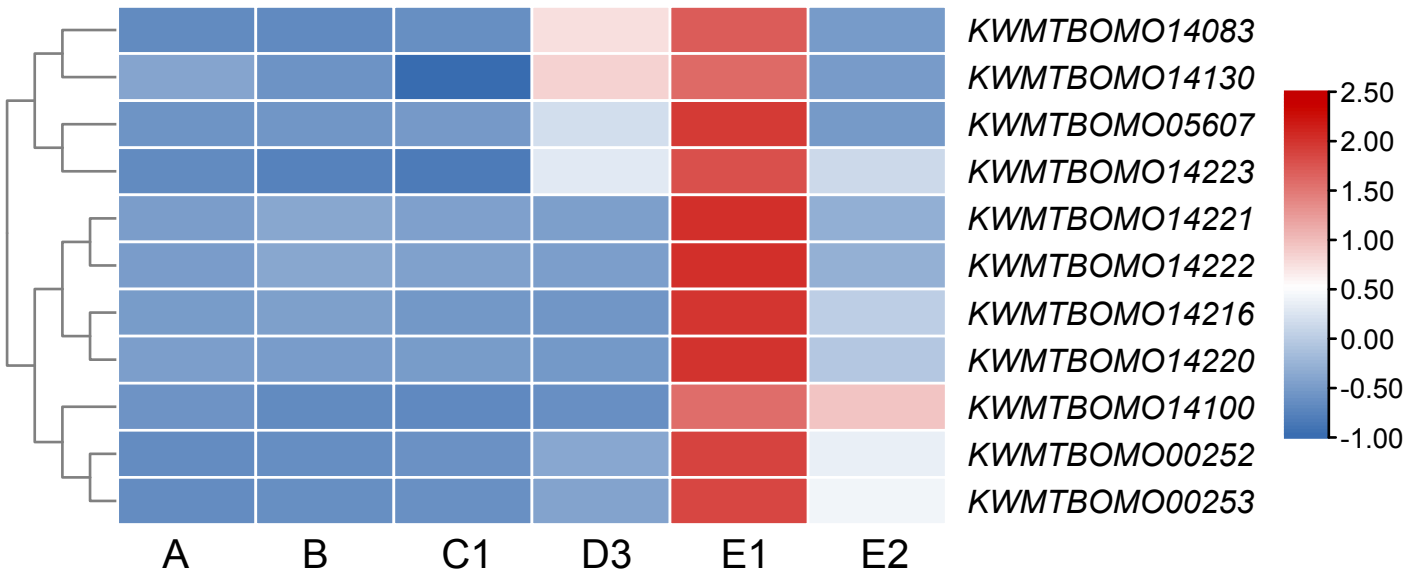

d

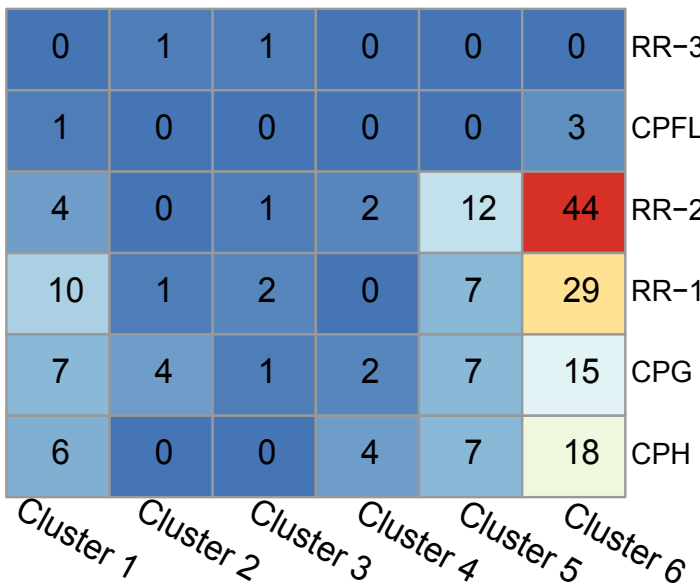

c

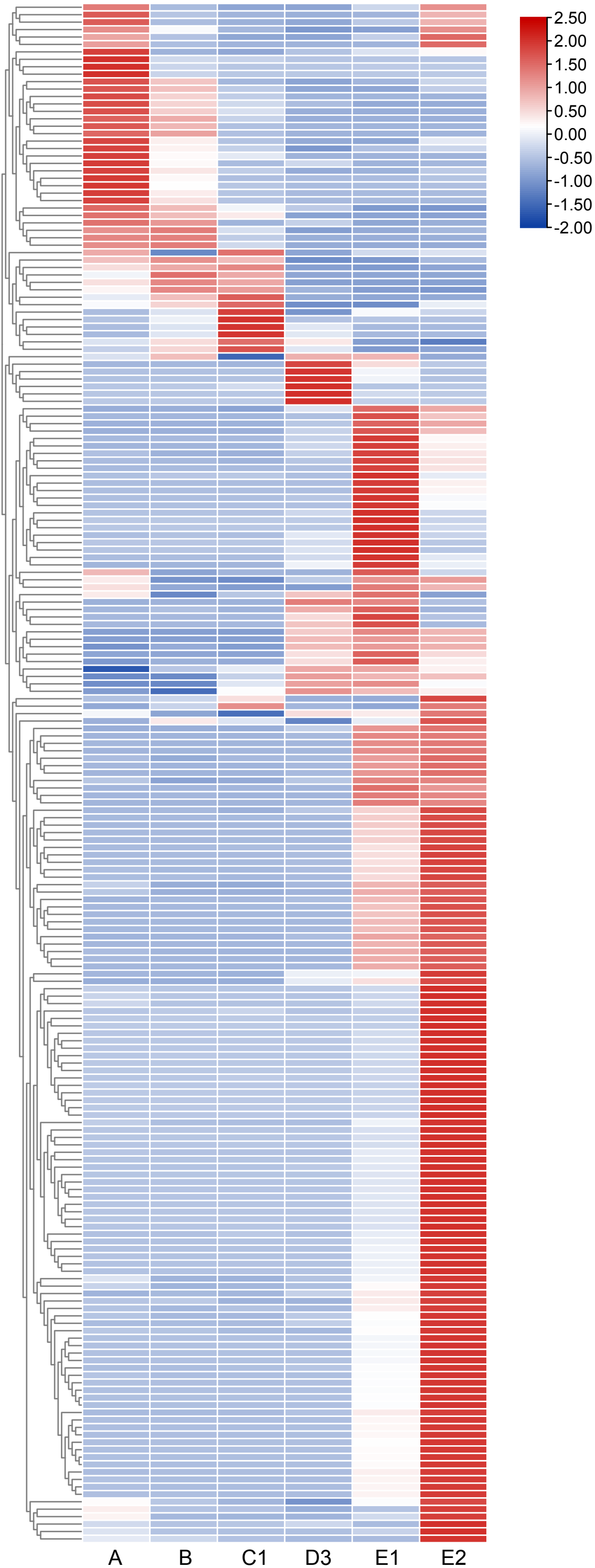

Supplement: S2 Fig — (a) Heatmap of chitin degradation (left panel) and biosynthesis (right panel) related genes. Different enzyme families were distinguished by color. (b) Heatmap of fatty-acyl-CoA reductases (FAR). (c) Heatmap of cuticular protein genes. (d) Numbers of cuticular protein genes belonging to different families were identified in specific expression cluster. (S2_Fig.PDF) [file pgen.1011837.s002.pdf]

Fig. S3

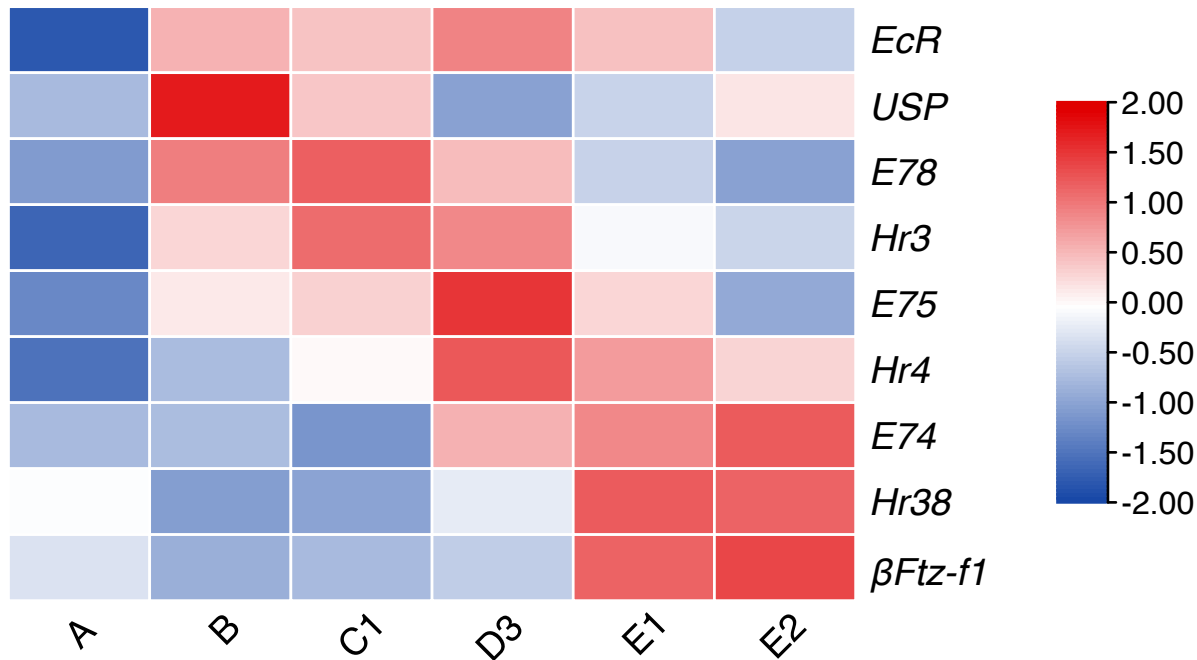

Supplement: S3 Fig — (S3_Fig.PDF) [file pgen.1011837.s003.pdf]

Fig. S4

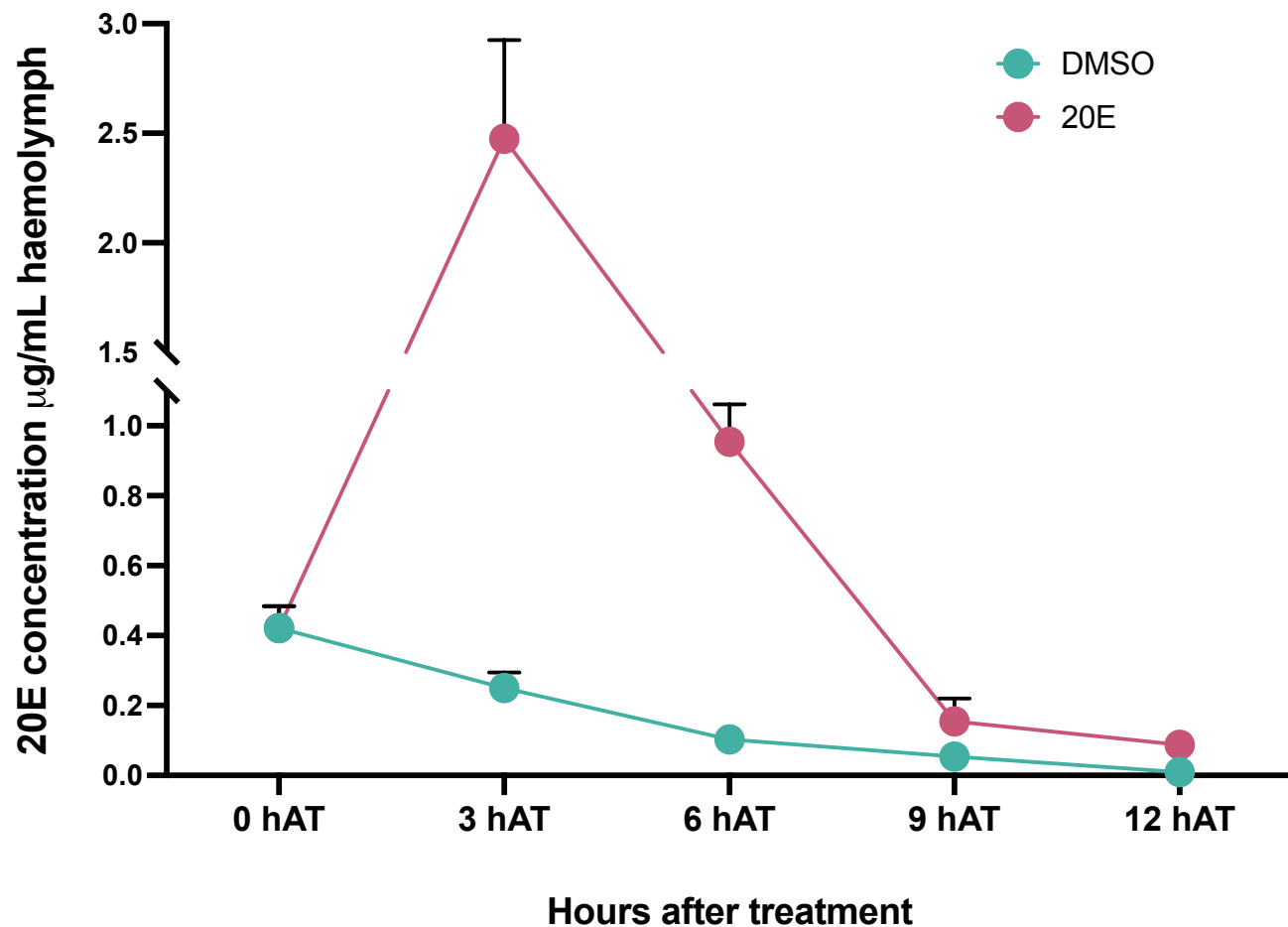

Supplement: S4 Fig — The larvae at D3 stage were injected with exogenous 20E or DMSO. The 20E titer was measured from the hemolymph collected at different time points. n = 3 biological replicates. (S4_Fig.PDF) [file pgen.1011837.s004.pdf]

Fig. S5

a

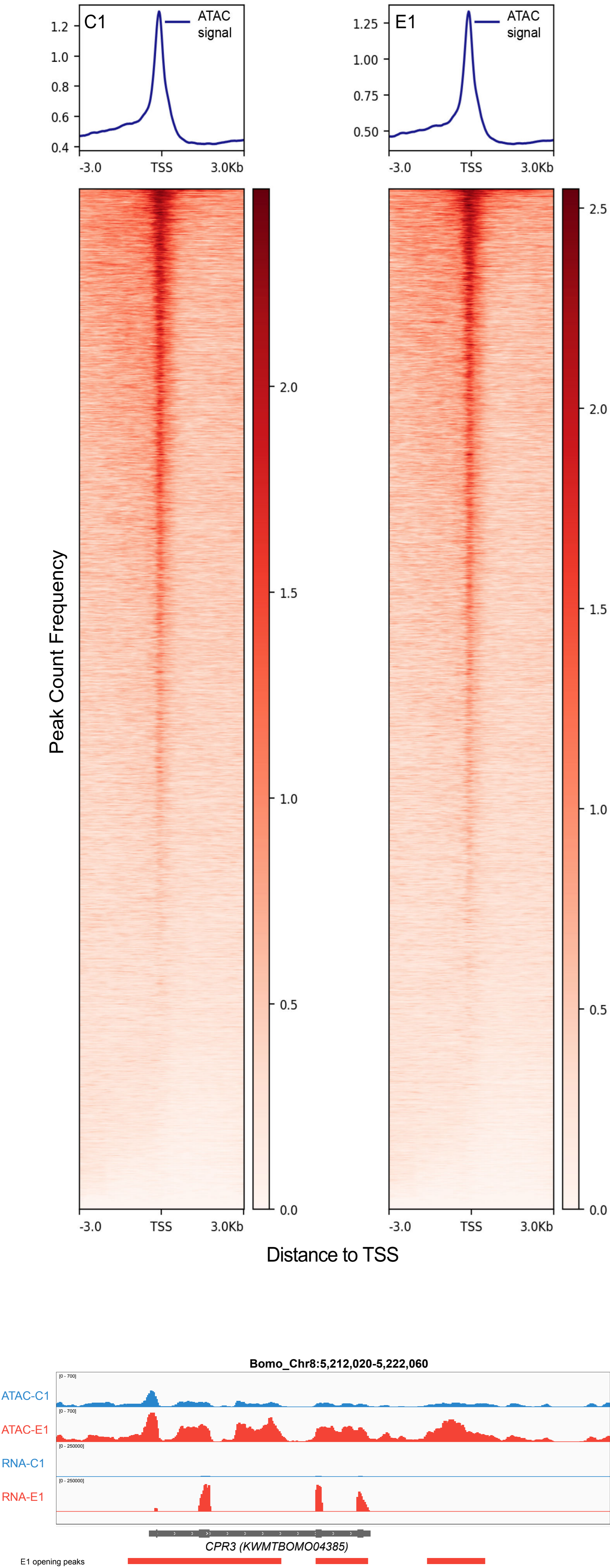

b

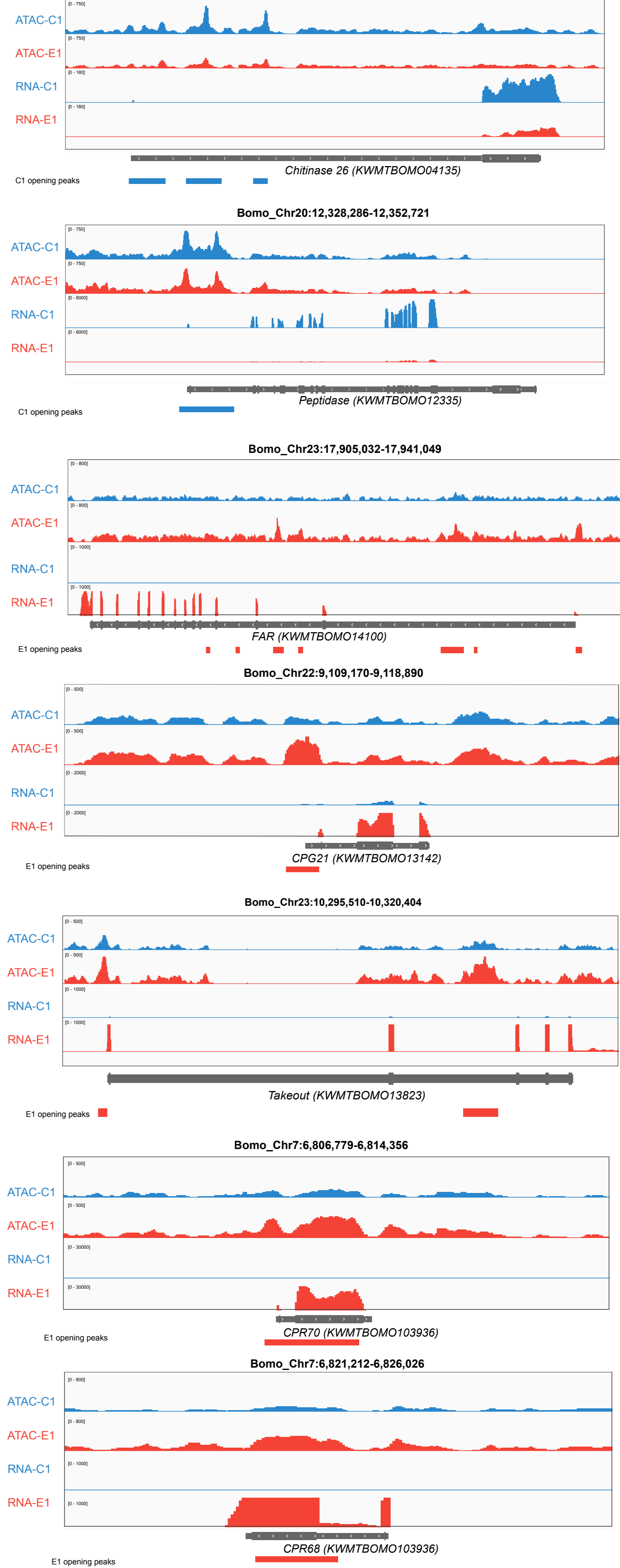

Supplement: S5 Fig — (a) Distribution of ATAC sequencing signals in the TSS of genes. (b) Genome browser shot of normalized ATAC and RNA sequencing counts of several represent genes. ATAC-seq and RNA-seq data from C1 stage in blue and data from E1 stage in red. Height indicates normalized ATAC-seq or RNA-seq signal. (S5_Fig.PDF) [file pgen.1011837.s005.pdf]

Fig. S6

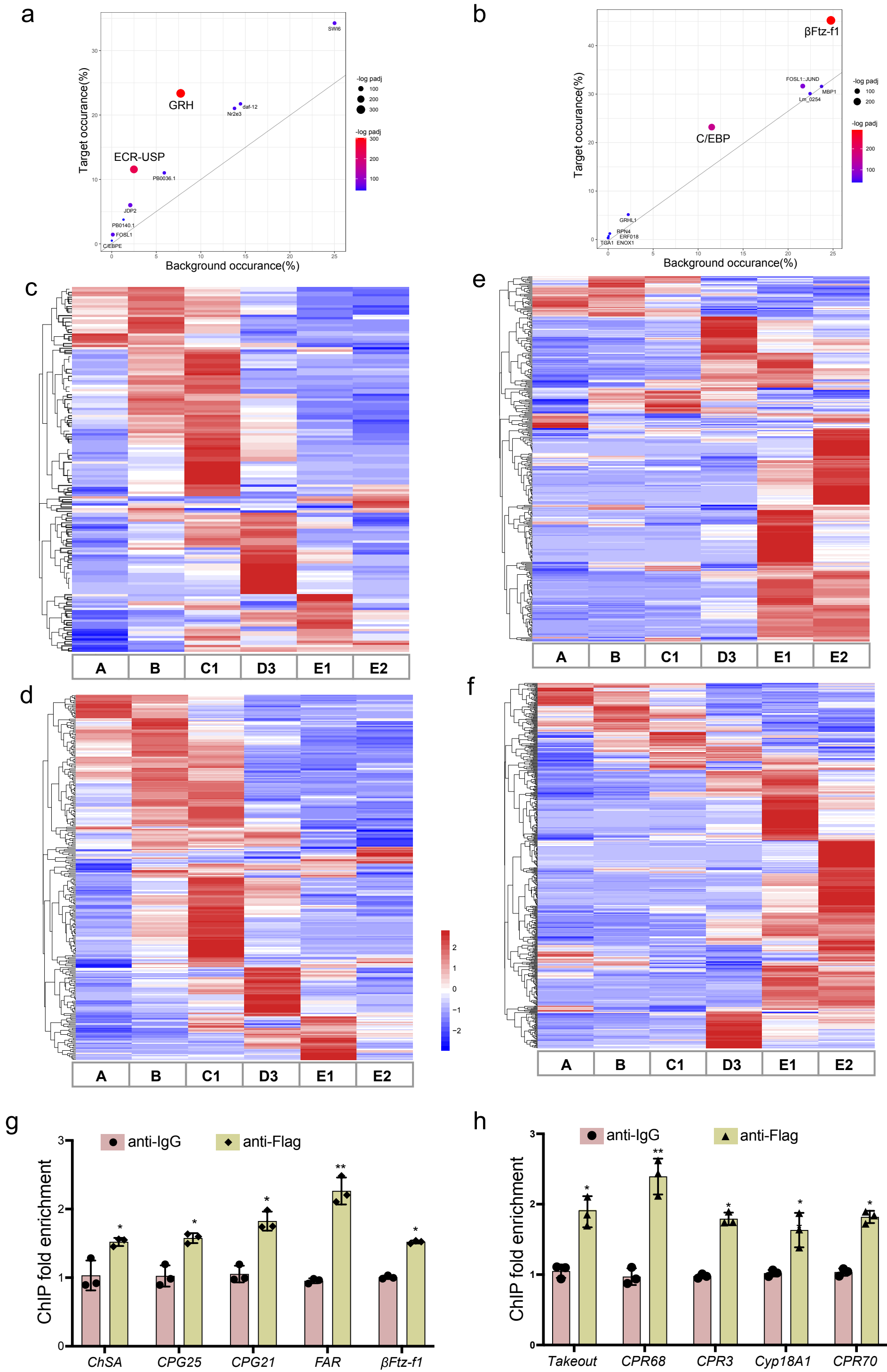

Supplement: S6 Fig — (a) Enrichment of TF motifs in ATAC-seq peak regions at C1 molting stage. “% of Targets” (y-axis) and “% of Background” (x-axis) represent the prevalence of a specific TF motif (spots) within open chromatin regions at C1 stage and genomic background, respectively. A statistically significant enrichment occurs when the “% of Targets” is significantly higher than the “% of Background”. (b) Enrichment of TF motifs in ATAC-seq peak regions at E1 molting stage. “% of Targets” (y-axis) and “% of Background” (x-axis) represent the prevalence of a specific TF motif (spots) within open chromatin regions at E1 stage and genomic background, respectively. (c) Heatmap of genes with ECR/USP binding sites. (d) Heatmap of genes with GRH binding sites. (e) Heatmap of genes with C/EBP binding sites. (d) Heatmap of genes with βFtz-f1 binding sites. (f) Quantitative real-time PCR from the ChIP assays in BmE cells that overexpressed Flag-tagged C/EBP. (g) Quantitative real-time PCR from the ChIP assays in BmE cells that overexpressed Flag-tagged βFtz-f1. (S6_Fig.PDF) [file pgen.1011837.s006.pdf]

Fig. S8  
a

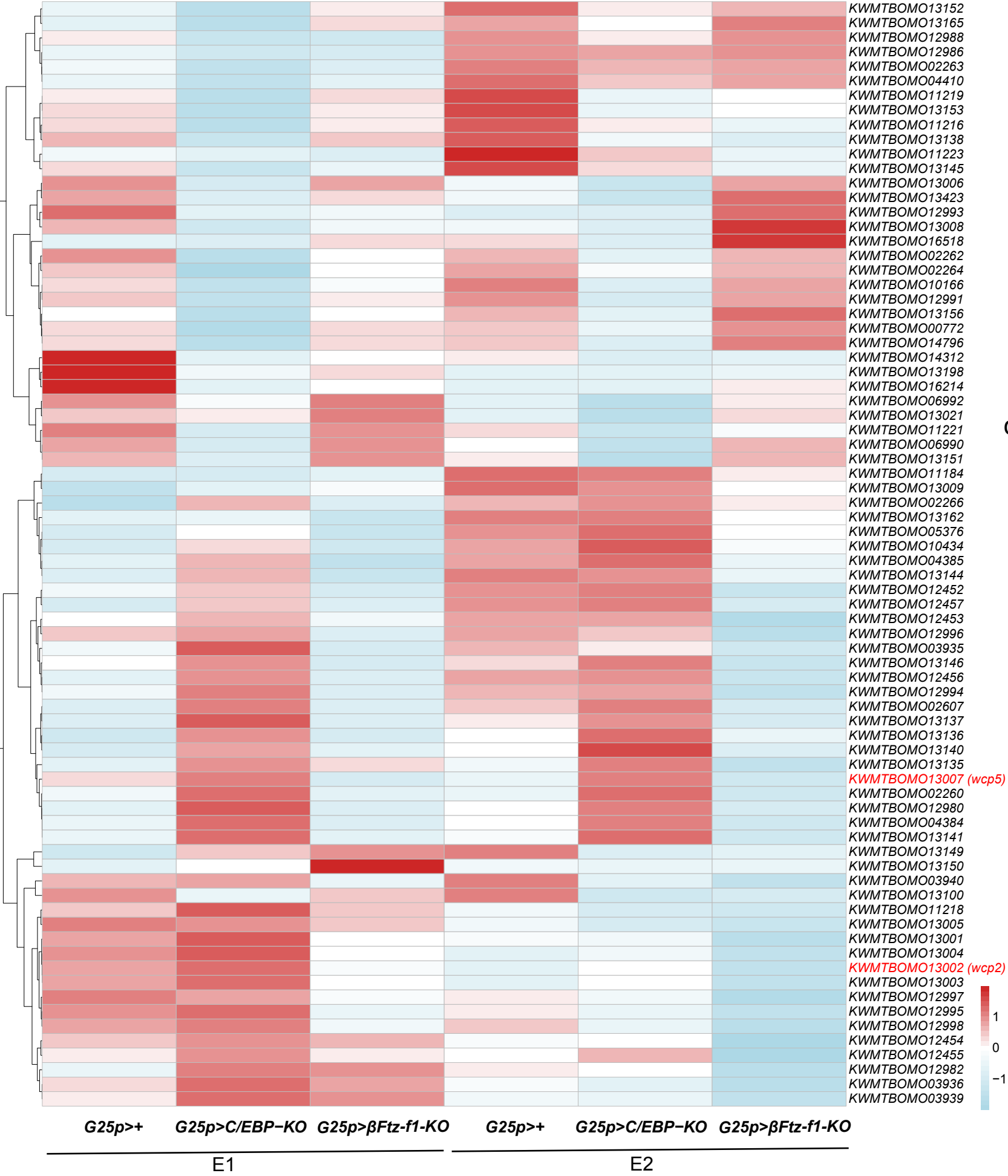

b

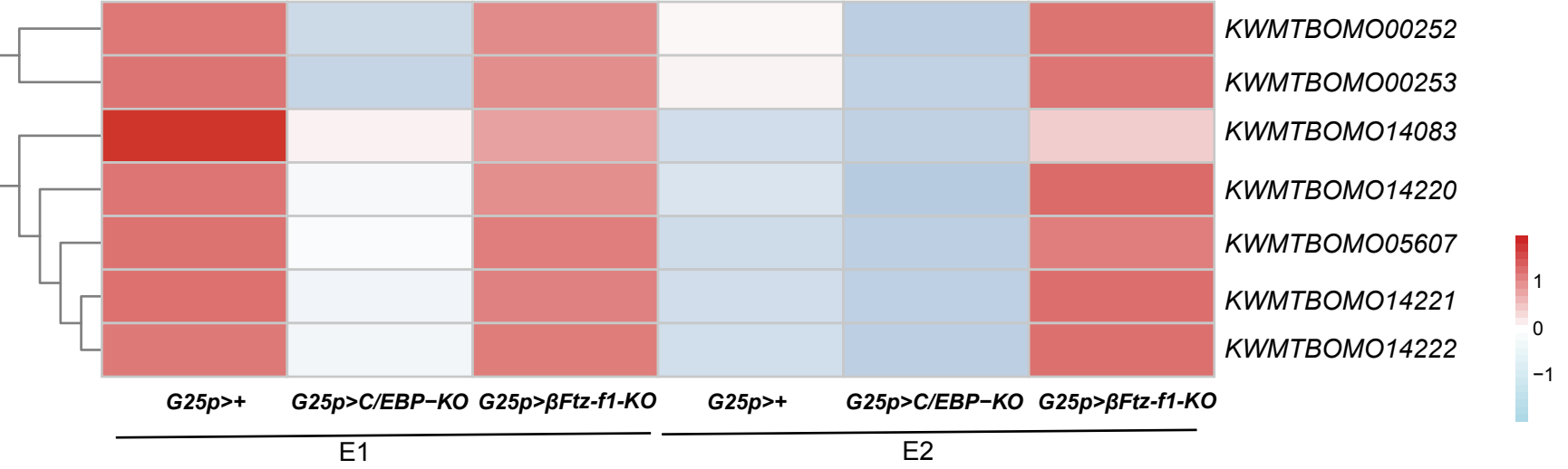

c

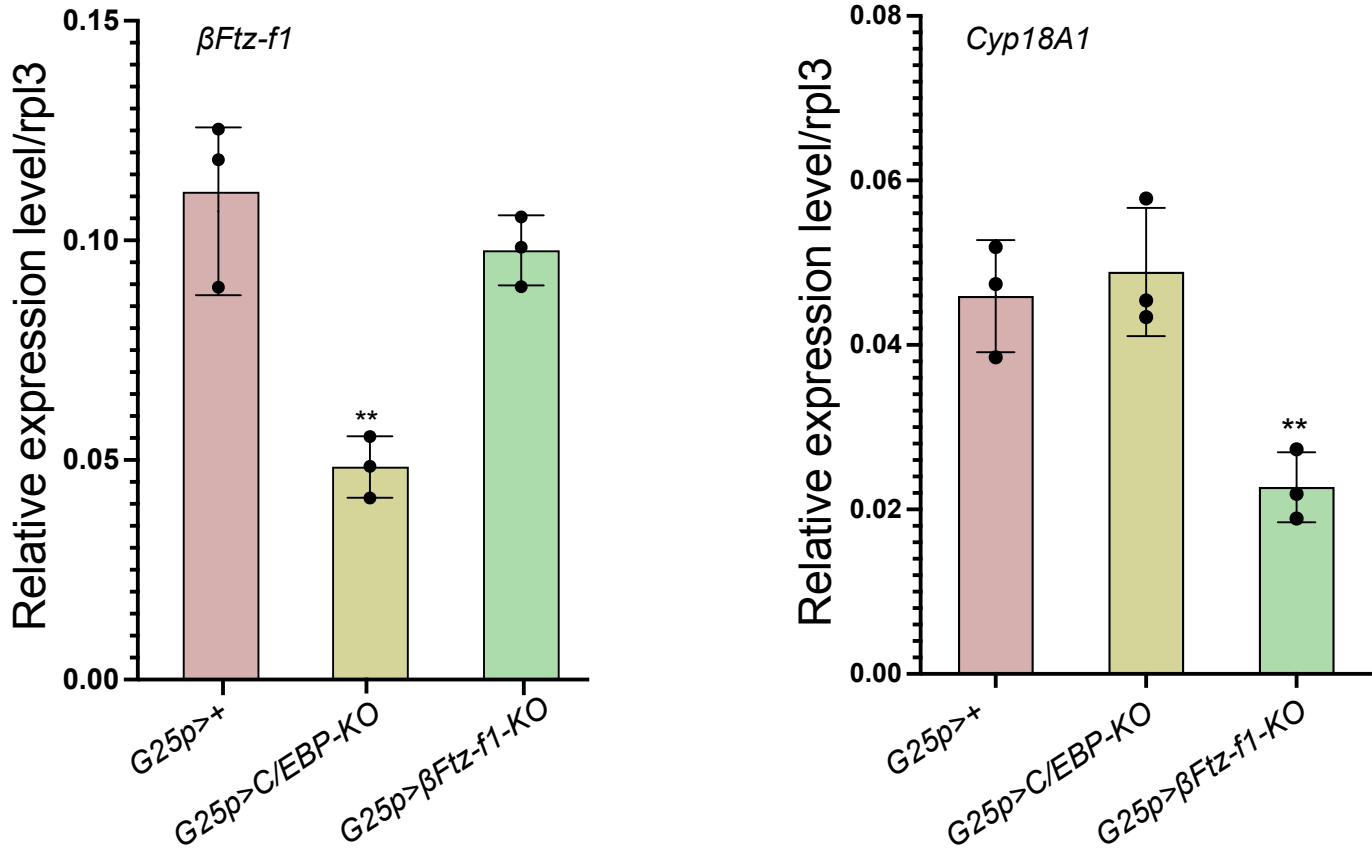

Supplement: S8 Fig — (a) Heatmap of differentially expressed cuticular protein genes identified in C/EBP and βFtz-f1 mutated larvae. (b) Heatmap of differentially expressed fatty-acyl-CoA reductase genes identified in C/EBP and βFtz-f1 mutated larvae. (c) The relative expression level of βFtz-f1 (left panel) and Cyp18a1 (right panel) gene in C/EBP and βFtz-f1 mutated larvae at 1st larval molting E1 stage. n = 3 biologically replicates. **P < 0.01. (S8_Fig.PDF) [file pgen.1011837.s008.pdf]
