# Supplementary material for: Transcriptomic landscape and chromatin accessibility uncover pivotal regulators driving programmed larval-larval molting in the domesticated silkworm
Source: PLoS Genet. 2025 Aug 19;21(8):e1011837. doi: 10.1371/journal.pgen.1011837 (PMC12380352; doi:10.1371/journal.pgen.1011837)
Supplement: S7 Fig — (a) The U6 driven sgRNAs transgenic silkworm with red fluorescent was crossed with epidermis specifically expressed Cas9 transgenic silkworm with green fluorescent, and their offspring with double fluorescent were selected to identify the genomic DNA mutagenesis. (b) the temporal expression pattern of Cas9 gene in Cas9 transgenic silkworm. The eggs and epidermis of 4th instar larvae were collected at indicated time points. Three biological replicates. (c) Mutant nucleotide sites of the C/EBP (upper panel) and βFtz-f1 (bottom panel) mutated larvae. The names with -egg means the sequences were amplified by genomic DNA extract from the egg. The names with -ref means sequence from silkworm reference genome, Other sequences were amplified by the epidermis from indicated mutated larvae as templates. (d) Mutation frequency of the targeting sites by amplicons sequencing. DF means the C/EBP or βFtz-f1 amplicons from the larva with double fluorescents. G25p means the C/EBP or βFtz-f1 amplicons from the larva with green fluorescent as wild control. (S7_Fig.PDF) [file pgen.1011837.s007.pdf]

Fig. S7

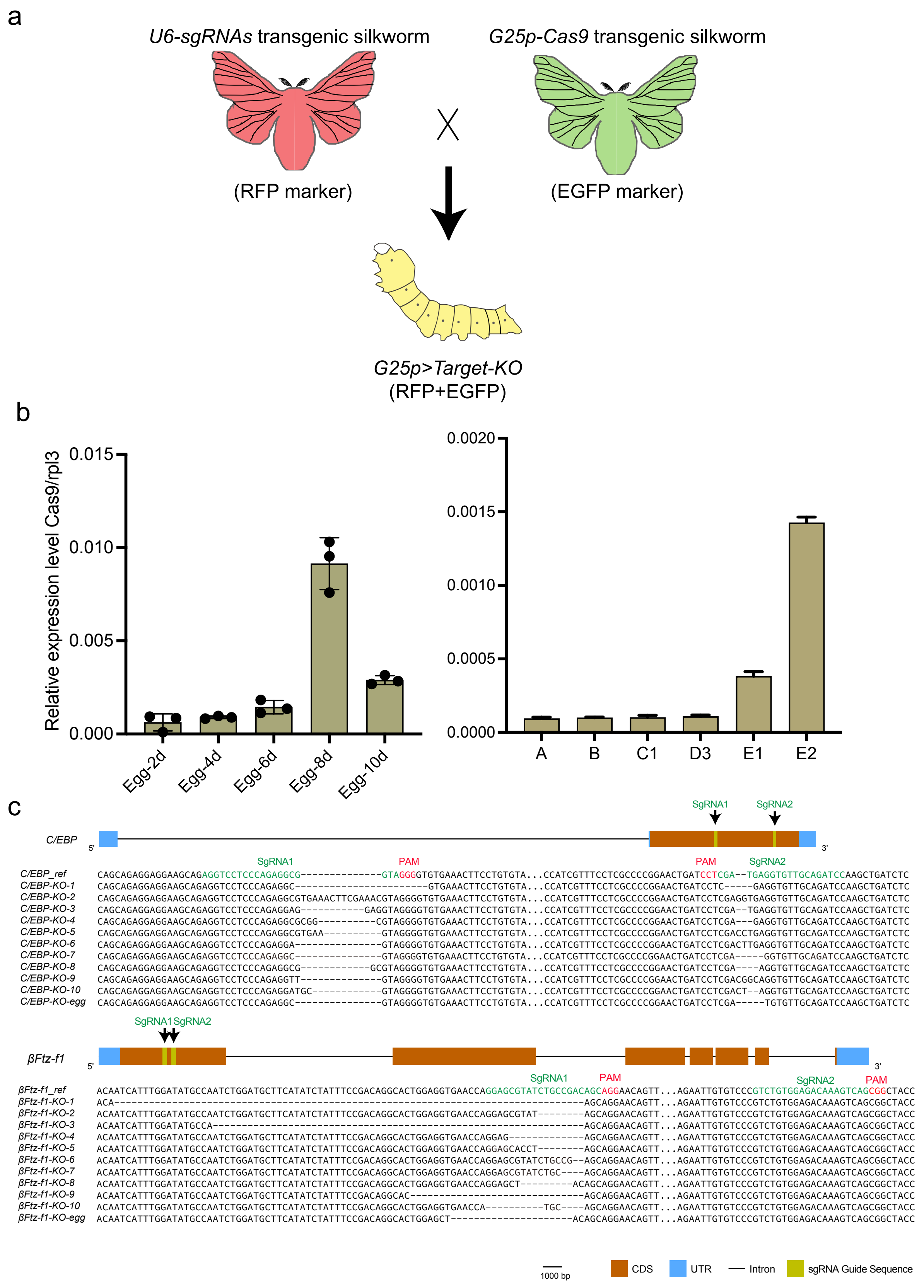

d

	Indel frequency	
	sgRNA1	*sgRNA2*
DF-C/EBP-1	49.4%	16.10%
DF-C/EBP-2	57.70%	30.30%
DF-C/EBP-3	58.50%	17.80%
G25p-C/EBP	0.00%	0.00%
DF-βFtz-f1 -1	97.20%	0.00%
DF-βFtz-f1 -2	97.50%	0.00%
DF-βFtz-f1 -3	97.30%	0.00%
G25p-βFtz-f1	0.00%	0.00%
