# Supplementary material for: Transcriptomic landscape and chromatin accessibility uncover pivotal regulators driving programmed larval-larval molting in the domesticated silkworm
Source: PLoS Genet. 2025 Aug 19;21(8):e1011837. doi: 10.1371/journal.pgen.1011837 (PMC12380352; doi:10.1371/journal.pgen.1011837)
Supplement: S1 Table — (S1_Table.DOCX) [file pgen.1011837.s009.docx]

**Supplemental Table S1. Primers list**

| Usage | Gene name | Forward primer (5’-3’) | Reverse primer (5’-3’) |
| --- | --- | --- | --- |
| ChIP-PCR | *ChSA (KWMTBOMO02041)* | TTTAGGCACGGATCTGTTAC | ATATGATTACGAAACTTTCG |
|  | *CPG25 (KWMTBOMO10666)* | GTACTGATGCTCCTCGCAGTC | TACTACTAACGTGGCATTGCA |
|  | *CPG21 (KWMTBOMO13142)* | GTGTGAAGTGGAGCCTCAAGC | CGACGAGGTCAAACCGATGAA |
|  | *FAR (KWMTBOMO14220)* | CAGGTTGGCAGTTCTATCATC | ACGTTTGAATCGAACGAGTGT |
|  | *βFtz-f1 (KWMTBOMO00379)* | GTGAGCGACCACGTTGGTAGA | GAAAGTGAGTCGGTGACCTAC |
|  | *Takeout (KWMTBOMO13823)* | ACGGGCCTAAAGCTAGCTCCAC | CAGCGGCGACATCGACTGCTA |
|  | *CPR68 (KWMTBOMO03939)* | CTTCTAAACGAATGTATGTCGTG | TTGTCCTTGACGAACAGTACCCTC |
|  | *CPR3 (KWMTBOMO04385)* | CGTCGTACCGTGGTATGTTG | ATGTTCAAAACGCCATCTGCG |
|  | *Cyp18a1 (KWMTBOMO05795)* | TTGATTACTTGCGAGTCTGGT | GAGCGCGTAGAATGTCGTTGA |
|  | *CPR70 (KWMTBOMO03936)* | GTACAGTTCATCGCTTCAAGCA | TTGGTCAGCATCAAGCTGTG |
| Real-time PCR | *C/EBP* | TCCCTTCGCCTTCTGTATTC | CACAGGACAATACAATCCGC |
|  | *βFtz-f1* | TCGGACTCTTACAAAGCCAA | CCATTGTGCATTCTCTGGTG |
|  | *RPL3* | GTGATGATCAAGGGTTGCTG | TCGCGAATACGATCCTTCTT |
| Transgenic plasmid construction | *C/EBP* | TGTGGTCTCTAAGTGAGGTCCTCCCAGAGGCGGTAGTTTTAGAGCTAGAAATAGCA^#^ | TGTGGTCTCGAAACCGATGAGGTGTTGCAGATCCACTTGTAGAGCACGATATTTT^#^ |
|  | *βFtz-f1* | TGTGGTCTCTAAGTGGGAGCGTATCTGCCGACAGCGTTTTAGAGCTAGAAATAGCA^#^ | TGTGGTCTCGAAACGTCTGTGGAGACAAAGTCAGACTTGTAGAGCACGATATTTT^#^ |
| Mutation detection | *C/EBP* | ACTCGACGAGCTCAATGGAC | GGTGCCGGTTTGTGTTTCAG |
|  | *βFtz-f1* | CAAGGCGCAGCATACCAATG | CTTTGCAGGATTCGCACGTC |
| Amplicons sequencing | *C/EBP* | *CCTACACGACGCTCTTCCGATCTACTCGACGAGCTCAATGGAC | *GTTCCTTGGCACCCGAGAATTCCAGGTGCCGGTTTGTGTTTCAG |
|  | *βFtz-f1* | *CCTACACGACGCTCTTCCGATCTCAAGGCGCAGCATACCAATG | *GTTCCTTGGCACCCGAGAATTCCACTTTGCAGGATTCGCACGTC |
| Reverse PCR | *PXL-IV1* | CAGTGACACTTACCGCATTGA | GACCGCGTGAGTCAAAATG |
|  | *PXL-IV2* | CGCTATTTAGAAAGAGAGAGCAA | TGTTATTTCATGTTCTACTTACGTGAT |

^#^ The red labelled DNA sequences represent sgRNA sequence; * The green lavelled DNA sequences mean adaptor sequence.
