# Supplementary material for: Transcriptomic landscape and chromatin accessibility uncover pivotal regulators driving programmed larval-larval molting in the domesticated silkworm
Source: PLoS Genet. 2025 Aug 19;21(8):e1011837. doi: 10.1371/journal.pgen.1011837 (PMC12380352; doi:10.1371/journal.pgen.1011837)
Supplement: Attachment — Submitted filename: Response to comments.docx [file pgen.1011837.s010.docx]

**Response to Editor’s Comments:**

In revising this manuscript, please pay particular attention to the comments of Reviewer 2.
Response: Thanks for handling our manuscript and providing comments. Following the comments of the three reviewers, we have performed some experiments to address the question, and revised the whole manuscript to make our description more clearly.

**Response to Reviewers' comments:**

**Reviewer #1**: In this manuscript, authors explain the results of transcriptome analysis comparing with morphological changes of the epidermis. They divided the stage into early, middle and late molting stages. Then, they divided genes into six clusters depending on the expression pattern. They examined the effect of 20E treatment. They operated ATAC-seq and ANA-seq analysis. Through the several bioinformatical methods, they found ECR/USP and Grainy head (GRH) motifs are two most prominently enriched in C1, and C/EBP and βFtz-f1 in E1. They examined the effects after treatment of hormone, the effect of KO of C/EBP and βFtz-f1, and speculated that C/EBP is a direct regulator to initiate the transcription of βFtz-f1. The information presented is valuable to readers, they found two prominent transcription factors in the early molting stage, and late molting stage respectively. However, the following points should be addressed in the revision:

#The consideration on the function of GRH is not enough. The reason of GRH motif enrichment and the possibility of its function should be more deeply discussed. They speculated that C/EBP is a direct regulator to initiate the transcription of βFtz-f1, but they did not show direct interaction of two, so their speculation should be changed. Explanation of Figs is too simple. I think authors should explain every figures more clealy.
Response: We sincerely thank for your valuable comment. We agree that our initial discussion of GRH's potential role lacked sufficient depth, particularly concerning its specific molecular functions within the context of our study. Although our data suggest that GRH binding motif is enriched during the early molting stage and previous studies have implicated it in epithelial development and chromatin accessibility, we acknowledge that drawing strong conclusions about its regulatory role in this specific context without functional evidence would be premature. Furthermore, we note that another reviewer independently raised a similar concern regarding the level of functional evidence presented for grh, specifically recommending a reduction in its discussion given the absence of direct functional validation. As our study mainly focuses on identifying and functionally characterizing key transcriptional regulators involved in the late stage of larval molting, we have toned down the statement of the *grh* gene in Discussion section of the revised manuscript. Please see the lines 440-450

Regarding C/EBP regulating βFtz-f1, our conclusion is based on these key results: 1) Our ATAC-seq data revealed a C/EBP binding site in the βFtz-f1 promoter region (Fig. 5i); 2) ChIP-PCR experiments confirmed C/EBP binds directly to that site (Supplemental Fig.S6e); 3) Knocking out *C/EBP* caused a significant drop in βFtz-f1 expression (Supplemental Fig.S8c). This strongly suggests C/EBP directly binds to βFtz-f1's promoter region and positively regulates its transcription. However, we agree our data doesn't prove C/EBP ‘initiates’ *βFtz-f1* expression – it's more accurate to say C/EBP is involved in regulating it. We've therefore revised the wording throughout the manuscript to reflect this more precisely.

According to your suggestions, we've expanded the explanations for the figures, adding more description and interpretation of the data to make them more clearly.

Minor comments are below.
Explanation of the number, C1, D3, E1, E2 is required.

Response: Thanks for your comment. According to previous study (Kiguchi and Agui, 1981. *J Insect Physiol*), the morphology change of spiracular (spiracular index) during the larval-larval molting process could be used as markers to represent different ecdysis events. To make the meaning of the numbers clearer, we have explained them in the M&M part of revised MS. Please see the lines 474-480.
Fig. 1a: illustrated ecdysteroid titer is required.

Response: Thanks for your comment. We have added the description and citation in Fig1 legend.
Fig. 1c: More explanation is need, PC1, PC2, PC3 are difficult to understand, and in the present, I think this figure should be deleted or more explanation need.

Response: According to your comment, we deleted Fig. 1c in the revised MS. Removing of this figure won’t affect the conclusion of our study.
Fig. 2b, BP, CC, MF; explanation is required here not in Fig. 7.
Fig. 2d, 2e: More explanation is required.
Fig. 2f, Explanation of colors and numbers are required.
Fig. 5a, b; More explanation is need.

Response: We are sorry for our limited explanation in our previous version. We have added more information to describe the figures and make them more clearly.
Fig. 7b, d: rich factor?
Response: Yes, it’s "rich factor" which quantifies the degree of enrichment of a specific GO term within a set of genes, typically those with differential expression. It's calculated as the ratio of the number of differentially expressed genes annotated to a particular GO term to the total number of genes annotated to that same GO term. We have added the annotation in Fig 7 legend.
L148: this stage, hormone increase?

Response: According to the report by Kiguchi and Agui (1981), the hormone concentration is increasing during the stages B and C1.
L189~: 20E titer in the hemolymph is ecdysteroid titer, not 20E.

Response: Thanks for your suggestion, we revised it to ‘ecdysteroid titer’. Please see the line 206.
L420; that, instead of the.

Response: Thanks for your suggestion, we revised the sentence to make it more clearly. Please see the line 397-401.

**Reviewer #2**: In this manuscript, the authors investigate gene expression dynamics and chromatin accessibility changes during the larval-larval molt in the silkworm Bombyx mori, using transcriptomic and ATAC-seq analyses.
While the methodological development in a non-model insect species is appreciated, much of the content looks descriptive.
It remains unclear to me whether the large-scale datasets generated in this study yield truly novel or impactful biological insights.


Major Comments:

1. Clarify novel versus known findings
The manuscript does not clearly distinguish between findings that are newly uncovered in this study and those that are reconfirmations of previously reported results.
These two categories should be clearly separated.
For example, Fig. 1a and 1b do not present novel findings.
Similarly, Fig. 2f reproduces a known result.
The authors should cite the relevant previous studies and clearly state that their data confirm these findings, thereby supporting the technical validity of their transcriptomic analysis.
Response: We thank you for this valuable comments. We agree that clearly distinguishing novel findings from confirmatory results is essential for contextualizing the study’s contributions. We will implement the following revisions to address this concern: **1)** Explicit labeling in maintext: In the Results and Discussion section, we added explicit statements to clarify whether our findings are newly identified or confirmatory of previous work. For example, in describing Fig.1a and 1b, we stated: “Consistent with the previous study…”, For Fig. 2f, and added statement to “these results reconfirmed a conserved transcriptional pattern of genes involved in the ecdysone hierarchy as in Manduca sexta” (Lines 218-220). **2)** Enhanced citations for confirmatory results: we added citations to the original studies reporting findings confirmed in our study. **3)** Reduced the reconfirmations data, to highlight newly uncovered data in our work, we have reduced the description of the reconfirmations data. For example, we reduced the statement about the expression profile of 20E responsive transcription factors shown in Fig.2f (We have moved this panel to supplemental Fig. S3) (Lines 211-217) and cuticle genes expression in Discussion section (Lines 372-376; 386-393) We hope these changes will emphasize the novel insights of our study while appropriately crediting precious studies. We appreciate your guidance on improving clarity.

2. GRH-related claims in the abstract
The authors describe GRH as a key early-stage regulator in the abstract, but no functional characterization of GRH is provided in the manuscript.
This statement appears to be an overinterpretation and should be toned down.

Response: We appreciate your insightful comment. We agree that our original wording in the Abstract or other part may have overstated the functional role of grh in the context of our study. To address this, we have revised the Abstract and Discussion section to tone down the statement and clarify that GRH is a putative early regulator, based on its conserved role in epithelial gene regulation and its binding motif enriched in our data. We now emphasize that, although functional characterization was not performed here, GRH remains a candidate for future studies investigating transcriptional regulation during the larval molting process. Please see the lines 440-450.

3. Use of the CPG25 promoter to drive Cas9
The authors use the CPG25 promoter to express Cas9, but this promoter is known to be active during the late E2 stage (Wang et al. 2022, IBMB).
Since C/EBP is expressed earlier, and bFTZ-F1 peaks around E1–E2, it is questionable whether this system is appropriate for analyzing these genes.
Moreover, the authors did not generate a knockout line or perform G0 somatic mosaic analysis—what was the reason for this choice?
Although induced mutations are reported, they should be treated quantitatively.
For example, the authors could separately extract genomic DNA from epidermis and non-target tissues (e.g., gut or fat body) and perform amplicon sequencing to assess mutation frequency.
Response: Thank you for your valuable comments. In our previous study (Wang et al., 2022. IBMB), we analyzed the spatial expression of *Cas9* mRNA and protein, finding that the nuclease is primarily expressed in epidermal cells, indicating tissue-specific functionality. However, that study did not examine the temporal expression pattern of the *Cas9* gene. Here, we investigated *Cas9* expression across different molting stages and found no expression during early molting, with high-level expression commencing from stage E1. Consequently, the temporal expression window of Cas9 overlaps with that of C/EBP and βFTZ-F1 (Fig. 6a, b; Supplemental Fig.S7b). Moreover, the signal of *Cas9* was also detected during silkworm late embryonic development (Supplemental Fig.S7b). Using genomic DNA from embryo with double-fluorescence (hybrid offspring of G25-Cas9 transgenic silkworm with RFP marker and U6-sgRNA transgenic silkworm with GFP marker) as template, the egg already harbors mutation in C/EBP and βFTZ-F1 genes (Supplemental Fig.S7 c, the sequence name labelled with ‘-egg’). Additionally, to quantify the efficiency of CRISPR/Cas9 system used in this study, we have sequenced more double-fluorescence individuals and detected various mutations at the target gene loci in all samples (Supplemental Fig.S7c). Thus, the CPG25-Cas9 system is suitable for studying C/EBP and βFTZ-F1 gene functions. Please see the lines 314-323.

Regarding knockout line construction, cuticle-specific knockout of these two genes resulted in complete lethality during early larval development (all died before the fourth instar). We also attempted ubiquitous CRISPR/Cas9-mediated knockout, which caused larval death at the first or second instar. Therefore, it’s hard to obtain knockout lines.
4. Epidermis dissection protocol
The dissection of the epidermis is not well described.
The authors should specify which body segments were used and clarify how thoroughly fat body and muscles were removed during tissue collection.
Response: Thank you for your valuable comments. We have added the method to dissect epidermis. For ATAC and RNA sequencing experiments, we only cut and collected the fresh dorsal integument of the segments 6 from 10-15 larvae. Then, under a stereomicroscope, attached fat body and muscle were carefully removed by forceps from epidermis. Please see the lines 498-500.
5. Discussion section is overly long
The Discussion is disproportionately long and at times redundant.
The authors are encouraged to streamline the section to focus on key messages and reduce unnecessary elaboration.
Response: We appreciate your constructive comments regarding the length and focus of the Discussion section. We agree that conciseness will enhance the clarity of our key messages. We have reduced some parts of description to focus on newly findings in this study. For example, we have deleted some sentences to describe the temporal expression pattern of cuticle protein genes which might be redundant in Results section. Moreover, we have reduced the description of grh gene.

Minor Comments:
Fig. 1b: "epidermis cells" → "epidermal cells"

Response: We sincerely thanks for your meticulous attention to detail. The error has been revised to “epidermal cells”
Line 126: "time serial transcriptome" → "time-series transcriptome" or "time-course transcriptome"

Response: Thanks for your comment. We have revised to time-series transcriptome

131: "B to D3" → possibly "C1 to D3"?

Response: We are sorry for the mistake. It should be “C1 to D3”
Line 148: "they were the hormone inducible" → "they were 20E-inducible genes"

Response: Thanks for your comment. We have revised to “they were 20E-inducible genes”
Line 176: The use of "more" is unclear, as it is not specified what factors are being compared.

Response: Thanks for your comment. We have changed “more” to “an”. Please see the line 192.
Fig. 1d: What does "Knkickkopf" refer to here?

Response: We are sorry for the mistake. It should be Knickkopf.

Line 189: "Hormone regulation of molting process" → "Hormonal regulation of the molting process"

Response: We are sorry for the mistake. We have revised to "Hormonal regulation of the molting process".
Line 335: "time-serial" → "time-series"Line

Response: Thanks for your comment. We have revised to time-series.

**Reviewer #3**: This MS contains some interesting results on the regulation of larval molting in the silkworm. Integrated analysis of RNA seq and ATAC seq data identifies new transcription factors involved in the regulation of expression of cuticular protein genes. Overall, the research is well designed, and appropriate approaches were employed, and results were interpreted well, and the conclusions are supported by the data included in the MS. Suggestions below are to help improve the MS.
1. The authors appear to use “molting” and “ecdysis" interchangeably, as well as “mRNA levels” and “gene expression”; please correct the manuscript to ensure proper usage.

Response: We appreciate your careful reading and valuable comment. We have revised the manuscript to ensure the precise and consistent use of terminology. Specifically, we now distinguish “molting” as the broader developmental process involving apolysis and the formation of a new cuticle, and reserve “ecdysis” for the specific act of shedding the old cuticle. Additionally, we have clarified our language by using “gene expression” when discussing transcriptional activity more generally. All instances have been carefully reviewed and corrected throughout the manuscript.
2. Please define abbreviations (e.g. EcR, GRH, C/EBP) used in the abstract and at first appearance in the text.

Response: Thank you for your valuable comments. We have carefully reviewed the manuscript and ensured that all abbreviations, including EcR (Ecdysone Receptor), GRH (Grainy head), and C/EBP (CCAAT/enhancer-binding protein), are now clearly defined upon their first appearance in both the abstract and the main text.
3.The Figures are crowded and therefore hard to get to the important point. I recommend moving some of the supporting Figures to supplementary data.

Response: We appreciate your comments regarding figure clarity. To improve readability and better highlight the key findings, we have reorganized the figures by moving several supporting panels to the supplementary section. For example, according to other reviewer’s comment, we deleted the PCA analysis in the Fig1. We have moved the enrichment of TF by ATAC sequencing in original Fig.5A to the supplemental Fig. S6. In addition, we have put the mutant nucleotide sites of C/EBP and βFtz-f1 mutated larvae in previous Fig. 6cd to supplemental Fig. S7. Corresponding figure legends and references in the text have been updated accordingly.
4. Line 51 frameworks are

Response: We are sorry for the mistake. We have changed to “are”.
5. Line 56, Endocrine mechanisms, hormones?

Response: Yes, it should be hormones.
6. Line 148, indicating that they are hormone inducible

Response: According to other reviewer’s suggestion, we have revised it to “they were 20E-inducible genes”
7. Line 138, genes with typical expression

Response: We are sorry for the mistake. We have changed to “typical”.
8. Line 197, As the hormone titers begin to increase (B stage), the transcript levels of ecdysone receptor (EcR) and its partner ultraspiracle (USP) also increased.

Response: Based on the comments from another reviewer, we have revised the presentation of this part to reduce descriptions of confirmatory findings from previous studies. Please see the lines 210-217.
9. Line 239, binding sites. Epidermis from two time points (Fig. 1a, C1 and E1), representing early and late molting stages, was selected for ATAC-seq

Response: We are sorry for the mistake. We have changed to “was”.
10. Line 246, showed that genes with high expression levels are distributed around the open chromatin

Response: Thank you for your comments. We have revised this sentence. Please see the lines 256-257.
11. Line 265, the expression profiles of the transcription factors were positively correlated with those of their target genes

Response: Thank you for your comments. We have revised this sentence. Please see the lines 274-276.
12. Line, 271, E1 stage, their targeted genes were enriched in new epidermal synthesis processes, such as structural constituents of cuticle, chitin binding, lipid biosynthetic processes

Response: Thank you for your comments. We have revised this sentence. Please see the lines 281-282.
13. Line 281, The two genes started to transcript from E1 stage indicating ecdysone-not clear, rewrite.

Response: Thank you for your comments. We have rewritten this sentence to make more clearly. Please see the lines 314-317.
14. Line 285, the hormone titers decreased to a level

Response: Thank you for your comments. We have revised this sentence. Please see the line 296.
15. Line 329, essential to the formation of new cuticle

Response: We are sorry for the mistake. We have deleted “.”.
16. Line 358 and throughout MS, replace Firstly with first.

Response: We are sorry for the mistake. We have replaced “firstly” to “first”.
17. Line 387, CPs from various families showed high and specific expression pattern during the late molting stages.

Response: We are sorry for the mistakes. We have revised this sentence. Please see the lines 386-387.
18. Please state the sources of chemicals and reagents used.

Response: Thank you for your comments. We have provided the sources of all chmicals and reagents used in this study.
19. Line 519, sequencing. Then the cells were lysed with cold lysis buffer to collect nuclei. Tn5 transposase was used to perform tagmentation of nuclei.

Response: We are sorry for the mistake. We have changed to “nuclei”.
