# Supplementary material for: Transcriptomic landscape and chromatin accessibility uncover pivotal regulators driving programmed larval-larval molting in the domesticated silkworm
Source: PLoS Genet. 2025 Aug 19;21(8):e1011837. doi: 10.1371/journal.pgen.1011837 (PMC12380352; doi:10.1371/journal.pgen.1011837)
Supplement: Attachment — Submitted filename: Response to comments_8-6.docx [file pgen.1011837.s011.docx]

**Response to Editor’s Comments:**

Please consider the comments of Reviewer 2 in making your revision.
Response: Thanks for handling our manuscript. Following the comments of the second reviewer, we have performed amplicons sequencing to address the question, and the revised part were all labeled with red.

**Response to Reviewers' comments:**

**Reviewer #1: Recommendation: Accept**

Response: Thank you very much for providing many constructive suggestions on our manuscript, which have significantly enhanced its quality.

**Reviewer #2: The authors have generally addressed my previous comments appropriately in this revision. However, there is still one point that remains unaddressed.**

Major Comments:
1. Analysis of the outcome of the transgenic CRISPR system
Previously, I requested a quantitative analysis of the mutant alleles generated by the authors' transgenic CRISPR system.
In the current revision, the authors only present the types of mutant alleles, but do not provide any data showing what proportion of the amplicons carry those mutations.
In an experimental design like this, where F1 individuals derived from crossing Cas9-expressing and sgRNA-expressing lines are analyzed, rather than fixed mutant lines, I think it is especially important to assess the frequency of mutant alleles quantitatively.
Whether the frequency is close to 100% or at an intermediate level (which may result in mosaic phenotypes) is crucial for evaluating the efficiency and reliability of the transgenic CRISPR system.
I strongly encourage the authors to perform amplicon sequencing using epidermal samples from both Cas9-sgRNA individuals and appropriate controls (e.g., sgRNA-only individuals), and to analyze the data quantitatively.
Importantly, the authors have already dissected the epidermis of L1 larvae produced via the transgenic CRISPR system and used them for RNA-seq and ATAC-seq analyses (Figs. 7 and S8).
Therefore, epidermis dissection in L1 is clearly feasible for the authors, and conducting amplicon-seq on those samples should be straightforward.
Finally, the raw amplicon-seq data should be deposited in a public repository.
**Response:** Thanks for your valuable comments. According to your suggestion, we have now performed amplicon sequencing on epidermal tissues collected from F1 progeny resulting from the crosses between Cas9-expressing and sgRNA-expressing lines, as well as from Cas9-only controls. The results are now presented in Supplemental Fig. S7d of the revised manuscript. Our sequencing analysis reveals clear differences in mutation frequencies across sgRNAs targeting different genes, and even between different sgRNAs targeting the same gene. For example, *C/EBP*-sgRNA1 induced mutations in approximately 55% of the amplicons, whereas *C/EBP*-sgRNA2 showed a lower efficiency of ~21%. The intermediate mutation frequencies observed for *C/EBP* sgRNAs suggest the presence of mosaicism in edited tissues. In contrast, *βftz-f1*-sgRNA1 exhibited a high editing efficiency, with >97% of the amplicons harboring mutations, while *βftz-f1*-sgRNA2 failed to induce detectable mutations. No mutations were detected in epidermal tissues from controls. The variability in editing efficiency among sgRNAs reflects well-known differences in sgRNA activity, such as sequence characteristics and chromain status of the target site (Konstantakos et al., 2022. Nucleic Acids Research). Importantly, our data confirm that the conditional knockout system itself is highly efficient when using validated sgRNAs (>97% in *βftz-f1*-sgRNA1 case). Please see the lines 321-328; 595-604. All the sequencing data have been deposited in to the National Genomics Data Center under BioProject accession code PRJCA032352.

We appreciate the reviewer’s suggestion, which has helped us to further strengthen our characterization of the CRISPR system and validate the reliability of our gene knockout strategy.
Minor Comments:
1. The short title is somewhat vague, as it does not clearly indicate what regulates the cuticle.
Clarifying the main subject of the study would help readers quickly grasp the focus of the work.

**Response:** Thanks for your valuable comment. We have revised the short title to “Transcription factor C/EBP and βftz-f1 regulate new cuticle layer formation during larval-larval ecdysis”. We hope this short title may better reflect the central focus of our study.

**Reviewer #3: I am satisfied with the revisions performed.**

Response: Thank you very much for providing many constructive suggestions on our manuscript, which have significantly enhanced its quality.
